# Long-term survival outcomes of pineal region gliomas

**DOI:** 10.1007/s11060-020-03571-z

**Published:** 2020-07-01

**Authors:** Joham Choque-Velasquez, Julio Resendiz-Nieves, Behnam Rezai Jahromi, Szymon Baluszek, Sajjad Muhammad, Roberto Colasanti, Juha Hernesniemi

**Affiliations:** 1 Department of Neurosurgery, University of Helsinki and Helsinki University Hospital, Helsinki, Finland; 2 Juha Hernesniemi International Center for Neurosurgery, Henan Provincial People’s Hospital, Zhengzhou, China; 3 Laboratory of Molecular Neurobiology, Nencki Institute of Experimental Biology, Warsaw, Poland; 4 Clinical Department of Neurosurgery, Central Clinical Hospital Ministry of Interior, Warsaw, Poland; 5 Department of Neurosurgery, University Hospital Düsseldorf, Düsseldorf, Germany; 6 Department of Neurosurgery, Umberto I General Hospital, Università Politecnica delle Marche, Ancona, Italy; 7 Department of Neurosurgery, Ospedali Riuniti Marche Nord, Pesaro, Italy; 8 Department of Neurosurgery, Helsinki University Hospital, Topeliuksenkatu 5, 00260 Helsinki, Finland

**Keywords:** Pineal region gliomas, extent of surgical resection, survival rate, long-term outcomes

## Abstract

**Purpose:**

Surgical series of pineal region gliomas are rarely available. Whereas it is a general assumption that the extent of surgical resection correlates with survival outcomes of intracranial gliomas; the impact of the microsurgical resection on the long-term outcomes of pineal gliomas has been questioned. We present a surgical series of pineal region gliomas with focus on the survival outcome analysis.

**Methods:**

17 histologically confirmed pineal region glioma patients classified as diffuse and non-diffuse gliomas were retrospectively analyzed. A detailed description of the series was followed by regression models to identify predictors of clinical outcomes. Uni- a multivariate survival analysis was performed to determine independent predictors of mortality.

**Results:**

Although the number of treated patients was small, only WHO grade histopathology remained significant (p = 0.02) after multivariate survival analysis with extent of resection, age, tumor volume, and preoperative functional status. The extent of the surgical resection did not correlate with the disease survival rates of non-diffuse (p = 1), diffuse (p = 0.2), nor all gliomas (p = 0.6). 15 of 17 patients underwent gross total (nine patients) or subtotal resection. The preoperative functional status of the patients showed overall improvement on the immediate (p < 0.001) and long-term (p = 0.03) follow-up after 106 (3 – 324) months.

**Conclusion:**

The extent of the surgical resection does not seem to significantly impact on the survival outcomes of pineal region gliomas. Thus, genotype and molecular features may essentially affect the outcome. Further research on the field is required.

**Electronic supplementary material:**

The online version of this article (10.1007/s11060-020-03571-z) contains supplementary material, which is available to authorized users.

## Introduction

Pineal region tumors represent around one percent of all intracranial neoplasms in the general population, and gliomas represent about 10% to 38% of pineal region tumors [1–8]. Gliomas are classified as diffuse and nondiffuse gliomas, and the recent 2016 World Health Organization (WHO) classification of tumors of the Central Nervous System (CNS) employed for the first time molecular features to subdivide gliomas, especially the diffuse ones [9, 10]. Pineal region gliomas originate from the pineal gland tissue or from the surrounding structures. Surgical series specifically dealing with pineal region gliomas are almost absent in the literature [7, 8]. Whereas it is a general assumption that the extent of surgical resection correlates with survival outcomes of intracranial gliomas; the impact of the microsurgical resection on the long-term outcomes of pineal gliomas has been questioned [8].

Our previous publication on the management of pineal region tumors reported an overall high correlation between a complete microsurgical resection and better survival outcomes [1]. Here, we aim to present and analyze the survival outcomes of the surgically treated pineal region gliomas in the department of Neurosurgery of Helsinki University Hospital (HUH).

## Methods

### Population study and design

This project has been approved by the Ethics Committee of HUH. This is a retrospective study of all consecutive histologically confirmed gliomas operated in HUH, between 1997 and 2015. Pre- and postoperative functional status measured by the modified Rankin Scale (mRS), and all the therapeutic modalities for the treatment were retrieved from hospital records. Radiological information of the gliomas was acquired from IMPAX 6.7.0.4511 (Agfa, Mortsel, Belgium). Final status of the patients was analyzed in July 2018. The extent of the surgical resection of the tumors was evaluated in the postoperative magnetic resonance imaging (MRI) studies. The term gross total resection (GTR) refers to the absence of any residual lesion in the postoperative T1WI, T2WI, or FLAIR MRI sequences. Subtotal resection (STR) means just a small residual lesion usually very attached to the neurovascular structures; and partial resection (PR) represents less than 95% of tumor removal. The American Academy of Pediatrics recommends 21 year-old as the upper age limit for the evaluation of pediatric patients [11, 12]. However, based on the distribution of our study population, we classified the patients as 5-year-old or younger and 20-year-old or older patients.

### Analysis of the data

R programming environment (© R Core Team, Boston, USA) was used for statistical analysis. We performed a general description of the study population categorized into 2 groups: the low-grade I-II nondiffuse glioma and the high-grade diffuse grade II-IV glioma groups. Initial uni- and bivariate analysis of the data was performed for identification of factors predicting clinical outcome. Fisher, Wilcoxon, or Spearman correlation tests were utilized wherever appropriate. Univariate survival analysis related with the diagnosis and with the extent of resection was performed by the likelihood ratio Cox model. The multifactorial survival analysis was determined by Cox survival model. The raw p-value cutoff for significance in all tests was set at 0.05. Adjustment of the p-value was performed with Benjamini–Hochberg procedure (α = 0.1).

## Results

Of the 76 surgically treated pineal region tumors, 18 glioma patients were operated during the study period. One patient with the histological diagnosis of gliosis/glioma was excluded from the analysis. A pilocytic astrocytoma (PA) patient was initially treated with biopsy and radiotherapy in 1985, followed by brachytherapy in 1995, and surgical removal in 2000. 17 patients (13 males) met the inclusion criteria: 10 nondiffuse grade I PA patients; two nondiffuse grade I-II ependymomas, and five diffuse (one WHO grade II, two WHO grade III, and two WHO grade IV) astrocytic gliomas. All of them underwent evaluation at the last follow-up (FU). All patients underwent surgery in praying sitting position by the senior author JH [13, 14]. Adjuvant radiochemotherapy protocols are detailed in Table 1.

**Table 1:** Adjuvant radiochemotherapy protocols for gliomas at HUH. Number of patients in parenthesis

	Adjuvant radiochemotherapy
Pilocytic astrocytoma (10)	A giant tumor was initially treated with biopsy and radiotherapy in 1985, followed by brachytherapy in 1995, and multiple surgeries since 2000. The patient died 324 months after initial treatment. No other patient received adjuvant therapy.
Grade I-II Ependymoma (2)	54 Gy (1.8 Gy/dia) after subtotal resection in one case.
Grade II Astrocytoma (1)	None, patient with complications died 3 months after complete resection.
Grade III Astrocytoma (2)	20 Gy (10 Gy/dose) of stereotactic radiosurgery and unspecified chemotherapy* for a small 5 mm local recurrence of the tumor, 15 months after subtotal resection (initially suspected as totally resected tumor). The patient died 52 months after initial treatment. Fractionated radiation therapy* and temozolamide 150 mg/m^2^ (unspecified number of cycles) after partial resection of the tumor. The patient died 12 months after initial treatment.
Glioblastoma multiforme (2)	54 Gy (1.8 Gy/dia) and temozolomide 150 mg/m^2^ (2 cycles) after subtotal resection of the tumor. More cycles of temozolamide were planned; however, the tumor progressed rapidly. The patient died 5 months after initial treatment. 36 Gy (3 Gy/dose) and temozolomide 150 mg/m2 (2 cycles) after complete resection of the tumor. More cycles of temozolamide were planned; however, the patient developed a pneumocystis infection and Eaton-Lambert syndrome. The patient died 9 months after initial treatment.

^*^Unspecified in the patient records

### Preoperative presentation and surgical features

The comparative analysis between the diffuse and nondiffuse gliomas is presented in Table 2. The clinical presentation of the patients was related with acute or progressive hydrocephalus symptoms (88%), visual and ocular detriment (41%), memory disturbances (24%), and motor deficits (12%). Detailed information of the patients is attached as appendix of this paper. The supracerebellar infratentorial approach was used in most of the patients. However, other approaches complemented multiple surgeries in two giant-PA patients. Around 50% of the patients underwent preliminary shunt for the treatment of hydrocephalus. An average of two additional shunt-related procedures per patient were observed in these patients. Two PA patients had five and 10 shunt-related surgeries. Some procedural complications were present in two patients of the diffuse glioma group (shunt infection, infarction and pneumocephalus); and in 5 patients of the nondiffuse glioma group (wound infections, meningitis, and pneumocephalus).

**Table 2:** Comparative analysis between the non diffuse glioma and diffuse glioma patients. For numeric variables, median (interquartile range)

	All *N:17*	Nondiffuse Glioma *N:12*	Diffuse Glioma *N:5*	P value	Adjusted p-value
Age in years	26 (20–51)	23 (4–33)	26 (21–64)	0.56	1
Sex: males	13 (76%)	9 (75%)	4 (80%)	1	1
Preoperative mRS (patients)	2 (1), 3 (3), 4 (7), 5 (6)	2 (1), 3 (2), 4 (5), 5 (4)	3 (1), 4 (2), 5 (2)	0.79	1
Hydrocephalus	15 (88%)	11 (92%)	4 (80%)	0.52	1
Preliminary shunt	8 (53%)	5 (45%)	3 (75%)		
Direct surgery	6 (40%)	5 (45%)	1 (25%)		
ETV	1 (7%)	1 (9%)	0		
Tumor size in mm					
Anterior–posterior	30 (25–38)	29 (26–55)	26 (21–35)	0.40	1
Cranio-caudal	22 (19–30)	26 (20–33)	20 (19–26)	0.64	0.9
Axial-wide	25 (22–36)	39 (25–50)	22 (22–36)	0.53	1
Surgical approach				1	1
SCIT	16 (94%)	11 (92%)	5 (100%)		
OTT	1 (6%)	1 (8%)	0		
Extent of resection					
GTR	9 (53%)	7 (58%)	2 (40%)	0.62	1
STR	6 (35%)	4 (33%)	2 (40%)	1	1
PR	2 (12%)	1 (8%)	1 (20%)	0.52	1
Immediate mRS (patients)	1 (4), 2 (6), 3 (4), 4 (3)	1 (3), 2 (4), 3 (4), 4 (1)	1 (1), 2 (2), 4 (2)	0.68	0.9
Follow-up in months	83 (52–148)	139 (136–231)	9 (5–12)	**0.002**	**0.011**
5-year survival	33%	100%	0	<** 0.001**	**0.005**
10-year survival	39%	100%	0	<** 0.001**	**0.006**
Final mRS (patients)	0 (3), 1 (6), 2 (1), 3 (1), 6 (6)	0 (3), 1 (6), 2 (1), 3 (1), 6 (1)	6 (5)	**0.004**	**0.016**

Values with statistical significance in bold

*ETV* external third ventriculostomy, *GTR* gross total resection *mRS* modified Rankin scale, *PR* partial resection, *OTT* occipital transtentorial approach, *SCIT* supracerebellar infratentorial approach, *STR* subtotal resection

### The postoperative follow up

The immediate postoperative evaluation showed a three-year-old WHO grade II glioma patient with poor neurological response after surgery due to tension pneumocephalus that required surgical decompression. Two diffuse glioma patients showed a new Parinaud´s syndrome and new double vision, respectively. Other two diffuse glioma patients did not present new neurological deficits. The immediate evaluation of the non-diffuse gliomas revealed four patients with new postoperative disfunctions (Parinaud´s syndrome, double vision with mild hemiparesis, and two patients with only new mild hemiparesis). At the last clinical evaluation, the diffuse glioma patients deteriorated progressively and died. One PA patient who underwent multiple surgeries harbored eye movement disorder associated with postoperative epilepsy controlled by medication. Another two-year-old PA patient who underwent primary radiotherapy and brachytherapy developed psychomotor developmental delay, right hand ataxia, and multiple meningiomas. The patient died 27 years after initial treatment with multiple surgeries for the tumor, for the shunt disfunctions, and for the treatment of the meningiomas. One patient had walking limitations after hip surgery unrelated with the neurosurgical procedure. All the other nine non-diffuse glioma patients harbored very minimal symptoms such as slight double vision (2 cases), sporadic headache (4 cases), or did not report symptoms (3 cases).

Two cases had early death within six months after surgery. The WHO grade II glioma patient died three months after surgery with an extensive delayed postoperative infarction in the territory of both posterior cerebral arteries. The patient underwent complete microsurgical resection of a large lesion (35 × 26 × 38 mm). The immediate postoperative imaging did not show signs of ischemia, but tension pneumocephalus that required neurosurgical decompression. The patient remained stable but with poor functional status. Two days later, MRI studies showed signs of infarction that increased in size for the following imaging. Another patient with a GBM that underwent a subtotal resection presented a rapid recurrence of the small lesion with diencephalic compromise in the MRI studies and died 5 months after surgery.

### Pre and postoperative functional status evaluation

**Fig. 1:**
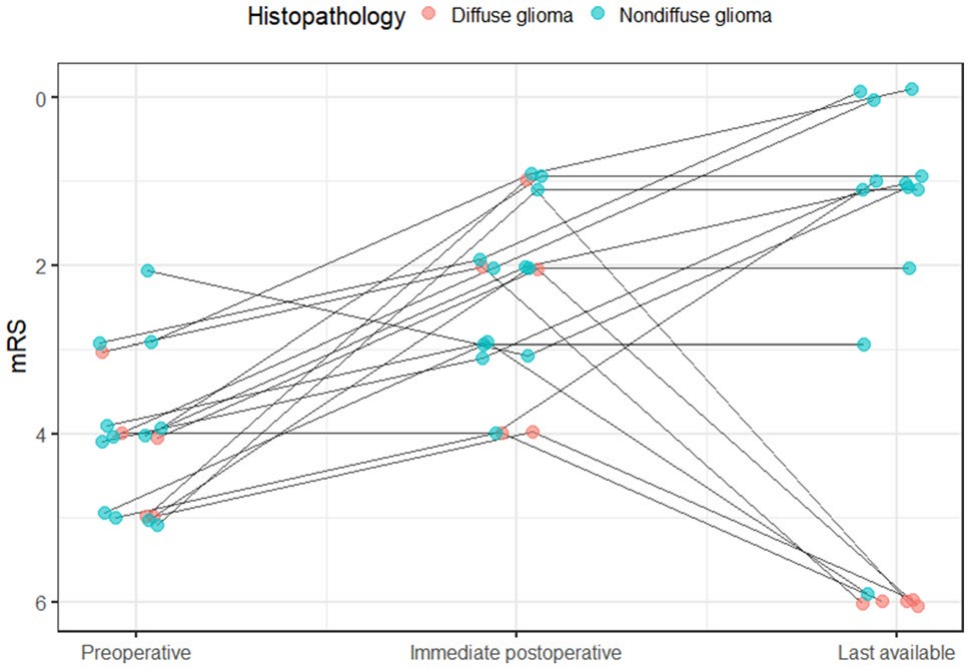
Preoperative, immediate (at hospital discharge of the patient), and last available postoperative functional status of nondiffuse low grade glioma (blue), and diffuse high-grade glioma (red) patients. mRS, modified Rankin Scale

The quantitative evaluation of the pre- and postoperative functional status of the patients demonstrated overall improvement at the immediate postoperative mRS measured at hospital discharge of the patient (Wilcoxon test, p = 0.0008) and last clinical FU (Wilcoxon test, p = 0.03) (Fig. 1). One patient with a mRS-2 at the last clinical evaluation underwent hip surgery before glioma diagnosis. The tumor size did not associate a strong correlation with the preoperative (Spearman test, R = -0.25, p = 0.34) nor postoperative (Spearman test, R = 0.15, p = 0.8) functional status of the patients.

### Mortality and extent of resection

**Fig. 2:**
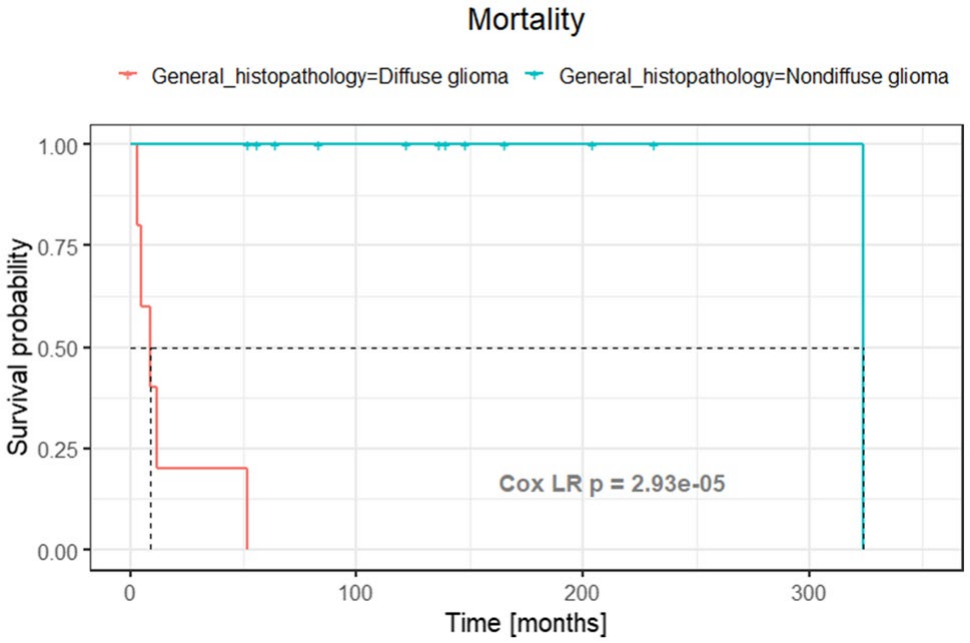
Kaplan–Meier curve comparing survival rates between diffuse and non-diffuse gliomas. p < 0.001 in the likelihood ratio Cox model (Cox LR). In dashed lines, 0.5 of survival rate

The overall mortality, similar to the disease-related mortality of this series, was established as 35.3% at a median (interquartile range) FU of 83 (52 – 148) months. The overall mortality for nondiffuse low grade gliomas was eight percent at a median (interquartile range) FU of 138 (78 – 175) months with a unique PA patient dead at 324 months from the initial treatment. All diffuse grade II-IV glioma patients died at a median (interquartile range) FU of 9 (5–12) months. The 5-year mortality was 100% for diffuse gliomas and 0% for nondiffuse gliomas (Fig. 2).

**Fig. 3:**
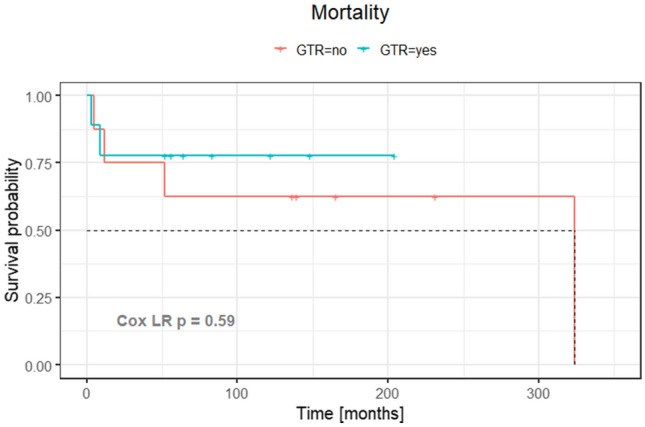
Kaplan–Meier curve for the analysis of extent of surgical resection and survival rates of pineal region gliomas; GTR, gross total resection. p = 0.6 in the likelihood ratio Cox model (Cox LR). In dashed lines, 0.5 of survival rate

Although the number of treated patients was small, the extent of the surgical resection did not impact on the disease specific survival rates of nondiffuse (p = 1), diffuse (p = 0.2), nor all gliomas (p = 0.6) under the likelihood ratio Cox model (Fig. 3). Moreover, the extent of the resection was similar among the different histological types of gliomas, and among the diffuse and nondiffuse gliomas (Table 2). Overall, 15 of 17 patients underwent GTR (six PA, one ependymoma, one grade II glioma, one grade IV glioma) or STR (three PA, one ependymoma, one grade III glioma, one grade IV glioma), and only two patients (the cystic component of a giant PA, and one grade III glioma) underwent PR. On the other hand, the evidence of tumor at the last MRI of patients who underwent incomplete resection was significantly higher than those who underwent GTR (Fisher test, p = 0.02). The extent of resection did not correlate with tumor size (Wilcoxon test, p > 0.05). However, tumors with median (interquartile range) large diameters of 30 (23 – 35) mm tended to GTR compared to those with median (interquartile range) large diameters of 35 (26 – 44) mm that underwent incomplete removal. None of the non-diffuse gliomas presented progression of the disease after surgery. The intracranial progression of the diffuse gliomas was as follows: The grade II glioma did not present recurrence. The two grade III gliomas presented local recurrences without distal recurrences. One grade IV glioma presented local recurrence without distal recurrence after subtotal resection; and the other grade IV glioma patient did not present local recurrence, but multiple foci of distal recurrence in the posterior fossa after complete resection of the tumor.

**Fig. 4:**
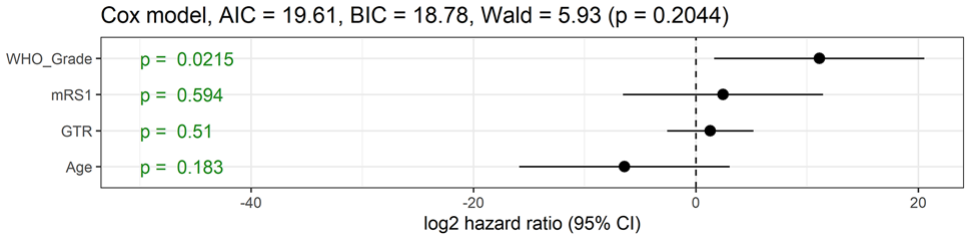
Multivariate Cox model analysis for the mortality of pineal region glioma patients. GTR, gross total resection; mRS1, preoperative modified Rankin scale; WHO_Grade, World Health Organization grade diagnosis

The best predictor of mortality in univariate Cox model analysis, the WHO grade of the tumor (p < 0.001), was the single significant variable (p = 0.02) after multivariate Cox model survival analysis with extent of resection, age, tumor volume, and preoperative mRS (Fig. 4). We could not properly incorporated adjuvant radiochemotherapy for multivariate analysis of mortality since adjuvant radiochemotherapy protocols of the gliomas, particularly the diffuse ones, widely varied between the patients (Table 1).

### Pediatric population

Details of pineal region gliomas in the pediatric population are presented in Table 3. Four children under the age of five (one female and three males) harbored gliomas (three PA and one grade II diffuse glioma) of the pineal region. The tumor size in children looked significantly higher compared to adults and might be focus of further research.

**Table 3:** Characteristics of the pineal region glioma in children. For numeric variables, median (interquartile range)

	All *N:17*	≥ 20-year old *N:13*	≤ 5-year old *N:4*	P value	Adjusted p-value
Age in years	26 (20–51)	33 (23–54)	2.5 (2 – 3.3)		
Sex: males	13 (76%)	10 (77%)	3 (75%)	1	1
Preoperative mRS (patients)	2 (1), 3 (3), 4 (7), 5 (6)	2 (1), 3 (2), 4 (5), 5 (5)	3 (1), 4 (2), 5 (1)	0.79	1
Hydrocephalus	15 (88%)	11 (85%)	4 (100%)	1	1
Preliminary shunt	8 (53%)	5 (39%)	3 (75%)		
Direct surgery	6 (40%)	5 (39%)	1 (25%)		
ETV	1 (7%)	1 (8%)	0		
Diagnosis				1	1
Non diffuse glioma	12 (71%)	9 (70%)	3 (75%)		
Diffuse glioma	5 (29%)	4 (31%)	1 (25%)		
Tumor size in mm					
Anterior–posterior	30 (25–38)	26 (23–30)	47 (37–64)	**0.006**	**0.048**
Cranio-caudal	22 (19–30)	20 (18–28)	31 (26–37)	0.06	0.32
Axial-wide	25 (22–36)	25 (22–30)	44 (37–51)	**0.003**	**0.048**
Surgical approach				1	1
SCIT	16 (94%)	12 (92%)	4 (100%)		
OTT	1 (6%)	1 (8%)	0		
Extent of resection					
GTR	9 (53%)	7 (54%)	2 (50%)	1	1
STR	6 (35%)	5 (39%)	1 (25%)	1	1
PR	2 (12%)	1 (8%)	1 (25%)	0.43	1
Immediate mRS (patients)	1 (4), 2 (6), 3 (4), 4 (3)	1 (4), 2 (5), 3 (2), 4 (2)	2 (1), 3 (2), 4 (1)	0.16	0.64
Follow-up in months	83 (52–148)	83 (52 – 148)	96 (40–185)	0.96	1
Tumor at the last MRI	47%	46%	50%	1	1
Overall mortality	35%	31%	50%	0.58	1
Final mRS (patients)	0 (3), 1 (6), 2 (1), 3 (1), 6 (6)	0 (2), 1 (6), 2 (1), 6 (4)	0 (1), 3 (1), 6 (2)	0.55	1

Values with statistical significance in bold

*ETV* external third ventriculostomy, *GTR* gross total resection, *mRS* modified Rankin scale, *PR* partial resection, *OTT* occipital transtentorial approach, *SCIT* supracerebellar infratentorial approach, *STR* subtotal resection

## Discussion

This is a report of the long-term outcomes of 17 surgically treated pineal region gliomas in HUH along 20 years of FU. Nondiffuse grade I-II gliomas had survival rates of 92%, while diffuse grade II-IV gliomas had 100% mortality along the FU. Differences in the extent of resection between GTR and incomplete resection -mostly represented by the STR- were not observed to consistently affect the survival outcomes of pineal region gliomas.

Tumors of the pineal region represent around one percent of all intracranial neoplasms with higher incidences in Northeast Asia. The distribution of pineal region tumors also widely vary between different populations with highly prevalent germ cell tumors in some Asia countries and pineal parenchymal tumors in some European countries [1, 3, 15–20]. Gliomas of the pineal region represent between 10 and 38% of the lesions [1–8]. According to the comprehensive review of Magrini et al., diffuse grade II astrocytomas are the most frequent subtype of tumor with 25% of the lesions, followed by glioblastoma with 19% of them [7, 8]. In this series, PA (59%) were the most frequent gliomas of the pineal region.

Gliomas of the CNS comprise two main groups, diffuse and nondiffuse tumors. The 2016 WHO classification of tumors of the CNS incorporated molecular features to subdivide gliomas. Diffuse WHO grade II-IV gliomas currently include genetic subgroups, such as diffuse glioma IDH-mutant 1p/19q codeleted, diffuse glioma IDH-mutant 1p/19q non-codeleted, and diffuse glioma IDH wildtype. Diffuse midline glioma, H3 K27M-mutant was also included in the last classification as a new entity with very poor prognosis. Moreover, other molecular features such as the TERT promoter mutation, the ATRX mutation, and TP53 among others, reorganized even more the diffuse gliomas. In regard to nondiffuse gliomas, PAs and ependymomas comprise the vast majority of them. Single abnormalities of the mitogen-activated protein kinase pathway are almost always present in PAs, revealing a possible single pathway disease. RELA fusion-positive ependymomas are very aggressive supratentorial tumors compared to the more benign grade I or grade II ependymomas [9].

While clear differences in the survival rates between low and high grade gliomas of the CNS are widely published [9, 10], some discrepancies still exist regarding the survival outcome of diffuse grade II astrocytomas of the pineal region. Even though several publications on the surgical management of pineal region tumors analyze gliomas together with other types of tumors, large series specifically dealing with pineal region gliomas are almost absent in the literature [7, 8]. A recent publication by Li et al. compared survival rates of low (WHO grade I and II) and high grade gliomas (WHO grade III and IV) of the pineal region. The mean survival rate of low grade gliomas (46% at a FU of 12.5 ± 11 months) contrasted with the mean survival rate of high grade gliomas (16% at a FU of 36 ± 23 months) [8]. The two diffuse grade II astrocytomas presented in that report were alive at 2 and 24 months of FU. On the other hand, the extensive review performed by Magrini et al. found that diffuse grade II astrocytomas of the pineal region have dismal differences in survival rates compared to the hemispheric counterpart, and recommended to classify gliomas of the pineal region in pilocytic and non-pilocytic gliomas [7]. In their series the two diffuse grade II astrocytomas died 1 and 12 months after partial resection and biopsy, respectively. In their words, the limited surgical resection of an infiltrative tumor placed in a deep surgical area seems to be a very important factor. Moreover, pineal gliomas could present molecular features of a diffuse midline glioma, H3 K27M-mutant, with very poor prognosis [21, 22].

The impact of the extent of the surgical resection in the long-term outcome of pineal region gliomas is not well established in the literature. Konovalov reported that the extent of resection was directly associated with survival rates of almost all types of malignant tumors of the pineal region [3]. However, even though this assumption could be right for pineal parenchymal tumors and non germinomatous germ cell tumors, diffuse high grade gliomas may not necessarily follow this tendency [1, 23]. Li et al. published a series of 25 cases of pineal region gliomas where differences in the survival outcome between tumors undergoing GTR and incomplete resection were not statistically significant [8].

Our previous report on the extension of surgical resection and the outcome of pineal region tumors gave as some clues about the reduced impact of the complete microsurgical resection of diffuse gliomas [1]. In this series moreover, we confirm that differences in the extent of resection did not consistently affect the survival outcomes of PAs nor low grade ependymomas. The long-term FU of the nondiffuse low grade gliomas discovered only one death of twelve patients at a median (interquartile range) FU of 138 (78 – 175) months, with five (42%) of them undergoing incomplete resection. The unique dead patient underwent partial resection with persistence of a cystic component after multiple surgeries for a giant 9 cm tumor. This patient died 324 months after initial radiotherapy and brachytherapy. One STR ependymoma patient received adjuvant radiotherapy with complete control of the tumor. Other three PA patients underwent STR without adjuvant therapy and remained stable along the follow up.

Obviously, the optimal surgical treatment of benign gliomas is represented by a complete removal of the lesions. However, in such large lesions with infiltrative tumor within the surrounding structures, an aggressive surgical behavior may determine bad clinical outcomes. In such cases, a subtotal resection of the tumors without the removal of small tumor tissues attached to vital neurovascular structures might offer a satisfactory result.

In regard to malignant lesions, the few cases presented here gave us a clue of their aggressive behavior independently of the extent of the surgical resection. Even though some series report some degree of survival in high grade gliomas, only proper long-term FUs with adequate registry systems will offer reliable results [7, 8]. The unique grade II astrocytoma in our series died 3 months after delayed thalamic infarction following GTR. A multiple extrapineal posterior fossa recurrence of a glioblastoma multiforme (GBM) followed probable cerebrospinal fluid dissemination after GTR, and a local recurrence of a grade III glioma appeared few months after GTR. It seems that genotype and molecular features of diffuse gliomas, such as the presence of H3 K27M-mutant, IDH wildtype, O-6-methylguanine-DNA methyltransferase methylation, or -according to last research- even excitatory synapse between neurons and cancer cells, are more important aspects to take into account for analyzing survival rates in diffuse pineal region gliomas [21–25]. Stowe et al. performed a comprehensive review of pineal region GBM and surprisingly discovered that GBM patients who received radiochemotherapy without tumor resection had better survivals than GBM patients who underwent resection and radiochemotherapy [23]. Obviously, more evidences are required to clarify in detail these findings.

The modest number of cases presented in this study is a clear limitation to generalize our results, particularly for high grade diffuse gliomas. Thus, Further studies with a bigger sample of patients are required to validate our hypothesis.

## Conclusion

In this small retrospective series, differences in the extent of resection between GTR and incomplete resection mostly represented by the STR, did not consistently affect the survival outcomes of gliomas of the pineal region. While diffuse gliomas carry a very poor prognosis. PA and low grade ependymomas have excellent long-term survival outcomes independently of the extent of resection.

## Electronic supplementary material

Below is the link to the electronic supplementary material.

Supplementary file1 (XLSX 11 kb)

## Data Availability

Data is available as a supplementary material for this manuscript.

## Abbreviations

CNS: Central Nervous System
FU: Follow-up
GBM: Glioblastoma multiforme
GTR: Gross total resection
HUH: Helsinki University Hospital
mRS: Modified Rankin scale
MRI: Magnetic resonance imaging
PA: Pilocytic astrocytoma
PR: Partial resection
STR: Subtotal resection
WHO: World Health Organization
